# Here and now: Lived experiences of professional nurses practising caring presence in a rural public hospital in the North West Province, South Africa

**DOI:** 10.4102/hsag.v25i0.1405

**Published:** 2020-08-05

**Authors:** Petronella S. Hobbs, Emmerentia du Plessis, Petronella Benadé

**Affiliations:** 1 NuMIQ Research Focus Area, School of Nursing Science, Faculty of Health Sciences, North-West University, Potchefstroom, South Africa

**Keywords:** caring presence, nursing presence, lived experience, descriptive phenomenology, rural public hospital

## Abstract

**Background:**

Practising caring presence is recognised as an important nursing intervention indispensable to high-quality, patient-centred care. An awareness of the real world of professional nurses (PNs) practising caring presence will assist in expanding and supporting the existing literature on the same. A clear and rich description of what the concept of caring presence entails within the unique South African nursing context may guide nurses in the art of this nursing skill, enhance their professionalism and facilitate the formulation of recommendations on how to encourage nurses to implement the practice of caring presence within nursing.

**Aim:**

This study explored and described the lived experiences of PNs practising caring presence in a rural public hospital.

**Setting:**

The study setting was a 120-bed, level-two district hospital in the North West Province of South Africa.

**Methods:**

A descriptive phenomenological method, specifically Husserl’s approach, informed this study. Semi-structured interviews were conducted with a purposive sample of 10 PNs. Data were coded and analysed using Colaizzi’s seven-step method.

**Results:**

Five themes emerged from the data analysis: professional caring presence, ethical caring presence, personal caring presence, healing caring presence and what caring presence is not.

**Conclusion:**

Professional nurses experience practising caring presence as professionally and personally fulfilling, as an expression of their passion for the profession, as a way of portraying ethical care, as a willingness to be personally present and as a healing experience that involves commitment and taking care of patients holistically. Unprofessional, unethical behaviour and depersonalisation of patients were indicated as uncaring behaviour.

## Introduction and background

The concept of caring presence is recognised as an extremely significant, valued core attitude in nursing practice, as well as a crucial element in quality healthcare (Kostovich 2012; Rowe & Kellam 2013). Bright (2012) assumed that the state of *being present* with someone in need characterises the practice of professional nursing.

According to Turpin (2014), caring presence is the competence of a nurse in enacting an inter-relational experience with a patient that produces positive patient outcomes. This capability is often equated to an individual’s ability to demonstrate the art of nursing practice.

The need for caring presence has been recognised worldwide (Valentine 2013; WHO 2014) and in South Africa also (Herselman, Le Roux & Opperman 2015). In this regard, Bright (2012) emphasised that when nurses practise caring presence in health institutions, these institutions are reformed in a profound and much needed way.

This study was undertaken in response to the challenge of exploring what it means to practise caring presence in the nursing profession. Caring presence is experienced internally and is thus difficult to fully describe, understand or enumerate (Turpin 2014). The concept of caring presence continues to retain a quality of mystery, even though it has been analysed and explored using several methods over half a century.

At the same time, clear and accurate knowledge regarding caring presence unique to *nursing* is becoming increasingly important. Rutherford (2012) made it very clear that the capability of nurses to create caring and effective moments and environments is of central concern in all healthcare settings.

To gain an in-depth understanding of the concept, caring presence can be explored from both the nurse’s and the patient’s viewpoints (Andrus 2013; Turpin 2014). The patient’s experience of presence is extremely important, as the main aim of caring presence is to create positive patient outcomes (Andrus 2013; Crane-Okada 2012; Kostovich 2012; Rutherford 2012). The exploration and description of professional nurses’ (PN) experience of caring presence are equally important, as this helps us to gain an in-depth understanding of the internal experience of the PN – grounding the need for research in this area. In addition, there is a need to develop interventions to promote caring presence, because relational and caring aspects in nursing are at risk (Boeck 2014; McMahon & Christopher 2011). New insights gained through this study may be used to guide nurses in the art of this nursing skill, enhance professionalism amongst nurses and highlight how caring presence can improve the quality of nursing care. This research enabled the formulation of recommendations on how to encourage nurses to implement the practice of caring presence within the nursing profession.

## Definition of concepts

### Caring presence

Caring presence is defined by Kostovich (2012:169) as ‘an intersubjective, human connectedness shared between the nurse and the patient’. For the purpose of this study, the researcher’s definition of caring presence is a connection to our own hearts to be felt by patients and nurses, which is enacted in special moments of *being there,* or *being with* another, in times of need. Caring presence has three levels: physical (body to body), psychological (mind to mind) and therapeutic (spirit to spirit) (McKivergen & Daubenmire 1994). It portrays the art of nursing within the nursing profession and is the gift of one’s self (Nelms 1996).

### Experience

Phenomenologically, Kisiel and Sheehan (2015) hold the view that in all of the psyche’s pure lived experience (in the perceiving of something, in the remembering of something, in the passing of judgement about something and in the willing of something), there is an intrinsic directedness towards something. Therefore, lived experiences are intentional, and they present to the individual what is true or real in his or her life.

### Professional nurse

A PN is a nurse who is registered with the South African Nursing Council (SANC) under Section 31 of the *Nursing Act* of 2005 (South Africa 2005).

### Purpose of the research

The purpose of this study was to explore and describe PNs’ lived experience of practising caring presence in the context of a rural public hospital in the North West Province, South Africa.

## Research methods and design

A descriptive phenomenological method, specifically Husserl’s approach, informed the design of this study. This approach entails a stance that asks ‘what do we know as persons?’ and is applied to carefully portray ordinary conscious experience of everyday life (Polit & Beck 2014), as was needed in this research. This approach is characterised by applying *epochè* (exploring taken-for-granted statements), bracketing and intuiting. In applying these principles, the researcher aimed to stay as close as possible to the data in their richness and complexity (Giorgi 1986:9). By utilising this method of inquiry, the essence of the PNs’ experience of practising caring presence within a rural public hospital in the North West Province could be captured.

### Setting

This study was carried out at a rural public hospital in the North West Province. The hospital mostly serves patients from remote and poverty-stricken areas. This 120-bed, level-two district hospital with an average bed occupation of 66% forms part of the public healthcare sector. This facility provides a comprehensive service, which includes operating theatres; a high-care unit; trauma and emergency care; neonatal unit; and maternity, medical surgical, gynaecological and paediatric wards. At the time of the research, the total number of nursing staff was 225, with a total of 59 PNs.

### Population, sampling and recruitment

For the purpose of this study, the target population (Grove et al. 2013) was PNs practising in a rural public hospital in the North West Province. A purposive sampling was used to select the participants. This sampling method was used to ensure that rich information regarding the lived experience of practising caring presence was obtained. The advantage of purposive sampling is that it allows the researcher to select a knowledgeable and representative sample group that is more likely to provide the requisite information about the phenomenon being studied (Brink et al. 2012).

The researcher involved a gatekeeper (Byrne 2016, Hill & Nutt Williams 2012) to gain access to the population. Within the context of this research study, the chief executive officer of the public hospital acted as the gatekeeper, who appointed unit managers as mediators to assist the researcher with the recruitment process. After permission was granted and the appointment of the mediators was confirmed, the researcher made an appointment with the mediators to explain the nature and purpose of the research. The mediators were requested to identify PNs who meet the inclusion criteria (see below) and who practise caring presence, based on behaviour such as not treating their patients as ‘a body in a bed’ but as a holistic person, who check regularly on patients, make eye contact, comfort patients, respond to the needs of patients, and portray interest and caring. The mediators were then requested to send an invitation and an informed consent document to all identified potential participants.

The sample was selected according to inclusion criteria. Participants were selected based on the criteria that they had to be PNs employed for at least 1 year in a rural public hospital in the North West Province, identified by a mediator as a PN who practises caring presence, and willing to participate.

Fourteen possible participants were identified through this process of recruitment and sampling, and 10 participants gave their informed, voluntary consent to participate. Data were collected until quality-rich data were generated and until the repetition of data was apparent (Burns & Grove 2011; LoBiondo-Wood & Haber 2010). Data saturation occurred when additional participants provided no new information and when themes that emerged became repetitive (Brink et al. 2012). After conducting 10 interviews, data saturation was reached in this study.

### Data collection

Semi-structured, face-to-face individual interviews were preferred as the means of data collection, for the rich data they provide, such as nuances of the participants’ experiences that may be conveyed through facial expressions, gestures, blushing or tears (Polit & Beck 2014). The data were collected by the first author, who was a master’s degree student at the time of the study. She completed a research methodology module and role plays in preparation for data collection. At the time of the study, she was working as a nurse manager at another hospital in the same region. Data collection took place during April and May 2017.

Two open-ended questions were asked to encourage participants to fully describe their experience (Welch 2015). The questions of the interview were the following:

- Can you please describe a situation where you practised caring presence as a professional nurse?
- How do you experience practising caring presence?

Each interview took about 45 min to 1 h. This included the time to create a rapport. The researcher recorded descriptions of the participants’ behaviours and demeanour during the interactions in the interview context by means of field notes to support the emerging themes.

### Data analysis

As participants were interviewed, the researcher reflected on their responses and made memos and notes. Following the interviews, the data were transcribed from the audio recorder to a Microsoft Word document by the researcher. The researcher engaged in prolonged immersion with the data whilst identifying and describing the true *essence* (or essential structure) of the experience (Gerrish & Lacey 2010). Transcripts were sent to an independent and experienced qualitative research co-coder. A confidentiality agreement between the researcher and the co-coder was utilised, and discussions were held to reach consensus on the findings.

Data were coded and analysed using Colaizzi’s (1978) seven-step method:

1. Each transcript was read and re-read in order to obtain a general feel of the content.
2. Each transcript was reviewed, and significant statements were extracted.
3. Each significant statement was reflected upon to formulate meanings.
4. The formulated meanings were organised into clusters of themes:
  - These clusters were referred back to the original transcripts for validation.
  - Discrepancies were noted amongst or between the various clusters, avoiding the temptation to ignore data or themes that did not fit.
5. Results were integrated into an exhaustive description of the phenomenon under study.
6. An exhaustive description of the phenomenon under study was formulated in as unequivocal a statement of identification as clearly as possible.
7. Participants were asked about the findings as a final validating step in order to compare the researcher’s descriptive results with their experiences. This step aimed to validate the study findings using the member-checking technique. Participants were provided written summaries of the findings (themes and sub-themes) and thereafter their views on the study findings were obtained via telephone calls. Minor revisions were integrated into the final description of the interviewee’s experience, but overall they agreed that the themes and sub-themes were a true reflection of their experiences.

From the transcribed interviews, 319 significant statements were extracted, leading to the development of 319 formulated meanings, reflecting the lived experience of these PNs. Eleven theme clusters were formed, which were further merged into five emergent themes.

During a training and information session, which was held in the boardroom at the hospital, the researcher conducted a PowerPoint presentation in which she shared with the mediators an introduction to the research activities, the purpose of the project, selection of the study population, as well as the methods and procedures by which data would be collected. Furthermore, she provided an explanation of the risks and benefits of the study, and confirmed the anonymity, voluntary participation and confidentiality of the participants. The mediators agreed to sign confidentiality agreements in order to protect the identity of the participants and to recruit the participants by sending an invitation to all participants meeting the inclusion criteria. The identified participants were given time (at least 24 h) to consider whether they wanted to participate. The voluntary consent was also confirmed prior to the audio-recorded, semi-structured interviews. Participants were ensured that they could withdraw from the study without any threats to their well-being at any time if they wished so.

The researcher assured that the transcripts and records were coded and numbered, and all data were kept confidential. Consequently, there were no links or clues to the identity of the participants. The audio recordings were destroyed by deleting them from the audio recorders after the transcribing process.

### Trustworthiness

Trustworthiness was demonstrated in providing rigour and strength to the study in accordance with the principles of credibility, dependability, confirmability, transferability and authenticity (Polit & Beck 2014). Strategies such as member checking, reflexive journaling, prolonged engagement, peer debriefing and enabling an audit trail were employed.

### Ethical consideration

Ethical considerations were adhered to in the following ways. The research proposal for the study was submitted and permission to pursue the study was obtained from the North-West University (NWU) Health Research Ethics Committee (HREC), Potchefstroom Campus (Ethics number NWU-00331-16-A1). In addition, the North West Department of Health as well as the chief executive officer (CEO) of the rural public hospital granted permission to conduct the research. The researcher utilised the informed consent form provided by the HREC of the NWU, Potchefstroom Campus. This consent form clearly stipulates the ethical principles of voluntary participation, beneficence, respect for people and justice.

## Findings and discussion

**FIGURE 1:**
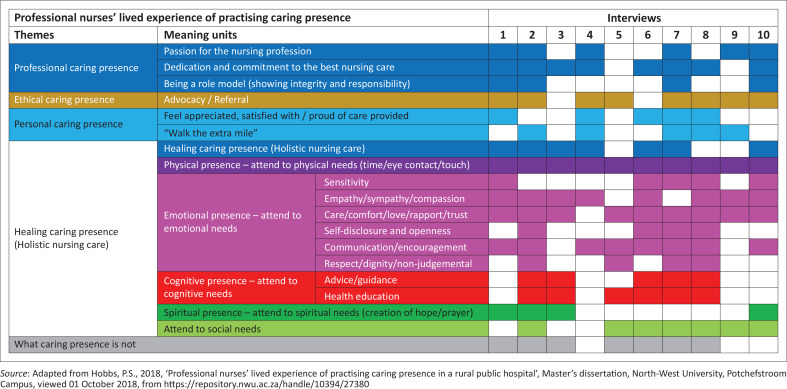
Coding table.

Table 1 outlines the demographic data of the participants who voluntarily participated in the semi-structured interviews. The five themes and sub-themes are illustrated in the final coding table, Figure 1, extracted from Hobbs (2018). In the discussion of the findings, quotes from the semi-structured interviews are provided as evidence, with the following key: T = Transcript, P = Page number, L = Line. The themes and sub-themes are discussed below in an integrative manner.

**TABLE 1:** Demographic profile of participants.

Number of participants	Age	Gender	Nursing qualifications	Work experience	Nursing unit
1	58	Female	Nursing Diploma	23 years	Neonatal
2	32	Female	Nursing Degree	10 years	Gynaecology
3	30	Female	Nursing Diploma	8 years	Theatre
4	27	Female	Nursing Diploma	5 years	HIV wellness clinic
5	31	Female	Nursing Diploma	9 years	Outpatients eye clinic
6	56	Female	Nursing Diploma	26 years	Outpatients eye clinic
7	31	Female	Nursing Diploma	8 years	Theatre
8	25	Female	Nursing Degree	3 years	Oncology
9	30	Male	Nursing Degree, Trauma specialised	8 years	Casualty
10	31	Male	Nursing Diploma, Theatre specialised	9 years	Casualty

*Source*: Adapted from Hobbs, P.S., 2018, ‘Professional nurses’ lived experience of practising caring presence in a rural public hospital’, Master’s dissertation, North-West University, Potchefstroom Campus, viewed 1 October 2018, from https://repository.nwu.ac.za/handle/10394/27380.

HIV, human immunodeficiency virus.

### Emergent theme 1: Professional caring presence

In their experience of practising caring presence, participants felt that *passion* is fundamental to the nursing profession, and the aspect of 100% *dedication* and *commitment* was emphasised. Therefore, they confronted the reality that a lack of self-awareness can result in a drop in professional care. Their experiences also included the practice of *leading by example*, showing integrity, enhancing professionalism and consequently portraying a caring attitude:

‘Yes, without passion, I could not do it … Passion goes for everything … You do it hundred per cent (silence). Everything comes with passion.’ (T1, P2, L48-50)

‘…I said to her, no, no, no, I am not here for the paperwork, I am here for the patients, you see.’ (T1, P4, L126-127)

‘We must be role models to the younger nurses to show them that we really care for our patients.’ (T 10, P53, L70-71)

‘The other thing, neh, is that you cannot nurse in a rural village, with a broken heart or with anger, it is so (silence) wrong.’ (T2, P12, L135-36)

Literature confirms that passion is the core characteristic that enables nurses to practise professional caring presence (Ketchem 2016; Wang 2017). McCaffrey (2012) also found that nurses who practise caring presence love being nurses and experience meaning from this role. Bigby (2015) and Jansen and Blair (2015:283) supported the participants’ views that understanding and having passion for the nursing profession establish professional nursing presence. Literature also highlights that professional presence involves the demonstration of compassion, respect, confidence, competence, integrity, optimism and passion (Wadsworth, Colorafi & Shearer 2017). Furthermore, research indicates that *self-awareness* is a dynamic transformative process of self, as well as a professional competency that facilitates and enhances the care experience (Bright 2012; Palmiery 2014).

### Emergent theme 2: Ethical caring presence

The participants described *portraying ethical care* as a way to practise caring presence. Therefore, they acknowledge patients as unique individuals and revealed a vibrant commitment to morally conducting nursing practice. They regarded the *advocacy* and *referral role* as important strategies to safeguard the best interest of patients:

‘But if I know I am fighting for my patients, their right to life, to get a chance.’ (T7, P42, L261)

‘Then I have to advocate for them. I beg them to give them a chance.’ (T4, P22, L59–60)

Ethical responsibility and moral sensitivity are particularly relevant to caring presence (Ray & Turkel 2015). Bright (2012) also found that caring presence results from a moral and ethical capacity, and nurses should have an ethical orientation towards connecting in a helpful and compassionate way with another human being. The importance of patient advocacy and defending the infringements of patient rights as highlighted by the participants is confirmed by Cole, Wellard and Mummery (2014) and Josse-Eklund et al. (2014). It was apparent in most participants’ experiences that there seems to be a strong connection between being a good nurse and ‘doing the right thing’, which supports virtue ethics (Bouchard 2016).

### Emergent theme 3: Personal caring presence

In their experience of practising caring presence, some participants revealed a desire to meet the *personal challenge* of being present, which requires a *true willingness* to become vulnerable. They felt *appreciated* through practising caring presence and were *proud* of the care they provided. They shared an intimate relationship with their patients by being personally available for them and treating them as if they were family members. These experiences were regarded as meaningful and enriching moments:

‘I try to become part of their family, or like a family member who cares genuinely. Yes, yes. I will go that far to come close to my patient.’ (T8, P49, L134–136)

‘It’s on a daily basis, neh, usually I am happy when I go home … I go home seeing a difference in the patients’ condition.’ (T1, P2, L53-55)

‘I feel I *walked an extra mile* for that patients, because remember … I gave everything.’ (T4, P23, L97-100)

‘I was actually giving my all for this patient. That is caring presence for me.’ (T7, P41, L226-227)

These findings reflect Palmiery’s (2014) statement that:

[A]s human beings, our presence is automatically care: it expresses the way in which we are, who we are, able to be, given our limits, and the context, both material and relational, in which we live. (p. 66)

When a nurse is personally present, compassionate care becomes real, and this state is needed for those who intend to facilitate healing (Welch 2015:93). Nurses who focus primarily on engaging in personal presence establish optimal milieus for intimate caring–healing interactions between the nurse and the patient (Sofhauser 2016). During shared moments of presence, PNs experience personal revitalisation, fulfilment and a sense of purpose (Trout 2013).

### Emergent theme 4: Healing caring presence

The participants regarded the practice of caring presence as an experience grounded in a *holistic nursing approach*. They shared dedication and willingness to *render patient-centred care* in order to establish a healing caring presence. Therefore, they revealed that caring presence is a conscious intention to focus on the total, holistic needs of patients:

‘Yes, to see the patient with physical, spiritual and emotional needs. Not only a body, but a person with more needs. Like I say to help the patient to heal.’ (T4, P24, L124-125)

‘All patients, sister, do have physical, emotional and spiritual needs, OK? I take care of all the needs and am present for the patient holistically.’ (T9, P53, L63-66)

‘I also take care of the patient’s spiritual needs. It is important to listen and if he needs to pray, let him pray and support him.’ (T10, P53, L56-58)

‘In rural areas, we have many social-economic problems. We must guide and assist the patients accordingly. It is very important.’ (T7, P45, L380-381)

‘Before you come to work, you must focus to be present. You must talk to yourself, you know what, today I am going to work for my patient, I am going to treat that patient that need me, because I am a nurse.’ (T2, P13, L144-149)

The nursing literature supports these experiences of the participants in similar ways, for example, the use of the self as an instrument of healing (McKivergen & Daubenmire 1994) and the gift of self (Osterman & Schwartz-Barcott 1996). Dossey and Keegan (2016) argued that powerful healing could be facilitated, even whilst engaging in task-orientated nursing activities, through caring presence. Furthermore, several studies support the finding that patient-centred care is an important principle that underpins caring presence (Kostovich & Clementi 2014; Mohammadipour et al. 2017). Kostovich (2012) confirmed that both being and doing, the essence of nursing presence, are reflected in individualised care. The practice of caring presence is considered to be an avenue that supports and fosters a healing environment (Bright 2012).

### Emergent theme 5: What caring presence is *not*

The participants further identified *barriers that hinder them to practise caring presence*. Stumbling blocks identified in this study were a shortage of adequate resources, a lack of time, a shortage of nurses and unbearable workloads, which result in poor nurse-to-patient ratios. Most of them strongly expressed themselves against uncaring role models, the negligence of patients, unethical nursing actions violating the rights of patients, as well as a lack of integrity in the nursing profession. Other participants warned against dehumanisation and depersonalisation of patients; they emphasised that patients should not be treated as objects nor called by their diagnosis:

‘In the ward, with thirty to forty patients, I have to give medication, I have to do vitals, I have to assist the patients. In the meantime I (am) needed with resuscitation. How are we able to cope?’ (T6, P34, L151-156)

‘What they are doing is, they sit with their phone (silence). It is WhatsApp (silence) or Facebook? Um (silence).’ (T1, P5, L147-149)

‘That the role-models also don’t care and that they are also on their phones … and they are also not there for their patients (silence). Even you can go for a lunch for 3 h (silence). Because the same manager goes for hours! And when she comes back, she just sits in the office. If you do this, they will follow you.’ (T1, P6, L193-194)

‘You must not call the patient by his diagnosis. You must say, Mr so and so and Mrs so and so. If you call them the laparotomy-patient, they don’t feel all-right. It is not fair to the patient.’ (T5, P29, L87-92)

Bright (2012) also found that the pressure on nurses to engage in supporting the system rather than the patient results in negative consequences for the nurses, such as guilt and shame, as well as the depersonalisation and humiliation of patients. Therefore, in agreement with what the participants shared, Van den Heever, Poggenpoel and Myburgh (2013) showed that nurses are often described as being ingenuine and insensitive in relation to patients’ true feelings. In addition, Iseminger et al. (2009) and Finfgeld-Connet (2006) support the argument that nurses experience a great deal of pressure to adapt to increasing workloads, growing nurse shortages and faster-paced healthcare systems. Therefore, these authors affirm that the modern healthcare system with its emphasis on productivity and high patient throughput poses a challenge to the ability of the nurses to practise caring presence for their patients.

## Strengths and limitations

Having selected a descriptive phenomenological design for this study, the researcher was able to incorporate the participants’ beliefs, insights, thoughts, actions and multiple realities regarding the practice of caring presence into an exhaustive description of the essence of their lived experiences. Furthermore, the semi-structured interview technique enabled the researcher to draw rich descriptions from the participants about the phenomenon of interest. In addition, the research findings were confirmed when literature integration was applied.

Through this research, the researcher gained insight into the description of specific and unique moments of this experience, thus making known the significance and transformative potential of caring presence in the nursing profession, for future use in the practice, education and research fields. Based on the findings, integrated with available literature, it could be concluded that the essence that represents the true nature of the phenomenon of practising caring presence in a rural public hospital shared by the participants regarding their lived experiences is:

[*a*] willingness and commitment to be professionally, personally and ethically present for and with patients in order to be a healing caring presence through rendering holistic, patient-centred nursing care. (Hobbs 2018:78)

The following limitations were identified in this study. Owing to the fact that this was a qualitative study, the research findings cannot be generalised to all PNs in the South African healthcare sector. However, the information captured the nuances of this lived experience as lived by these PNs practising caring presence in the context of a rural public hospital. The study was conducted only at one rural public health facility in the North West Province, thus limiting the study’s findings to this specific setting and to the PNs. The study reflected the lived experiences of only 10 participants, identified by the mediator, who volunteered to participate in the study. Data saturation was, however, reached. Owing to the high workloads of the participants, the urge to finish the interview as soon as possible in order to return to their units was observed by the researcher, even though data saturation was obtained.

## Recommendations

The recommendations focus on enhancing and encouraging the practice of caring presence in the nursing profession. Therefore, based on the findings and conclusions of this study, the following recommendations are made.

### Recommendations for nursing education

- Curriculum planners in charge of the degree programme should give more emphasis to the practice of caring presence in the nursing profession so that the value and transformative potential of the practice of caring presence can be understood by students to ensure patient-centred quality care and professionalism.
- Educators should give more emphasis to the importance of self-awareness and the therapeutic use of the self in the training of nurses.

### Recommendations for nursing practice

**Regarding the first emergent theme: Professional caring presence**

- A vibrant passion for the nursing profession should be cultivated and maintained so that nurses can come to practise caring presence and build meaningful relationships with patients.
- Professional nurses should portray professionalism and competence.
- Professional nurses should engage in continued professional development by means of workshops, in-service training and motivating courses. In this way, they can become role models to enhance the practice of caring presence, professionalism and a caring attitude amongst all nursing categories.
- Professional nurses should realise the importance of leading by example for other nursing categories to facilitate the practice of caring presence, professional attitudes and behaviour.

**Regarding the second emergent theme: Ethical caring presence**

- Ethical awareness and moral responsibility towards patients can be encouraged by means of courses and workshops.
- Professional nurses can facilitate and encourage the importance of patient advocacy and referral.
- Professional nurses can facilitate caring presence by being role models to other nursing categories, in portraying good, ethical conduct and showing genuine interest in the well-being of their patients.

**Regarding the third emergent theme: Personal caring presence**

- A willingness to be personally available, to walk the extra mile and to offer the gift of self should be recognised and encouraged by organisational management.
- The provision of workshops and in-service training regarding the significance of being personally present for patients can enhance the practice of caring presence in the nursing profession and thereby improve the quality of nursing care.

**Regarding the fourth emerging theme: Healing caring presence**

- Dedication and commitment to take care of patients holistically and to render individualised, patient-centred nursing care should be part of hospital policy.
- Awareness of the dimensions of being a healing caring presence can be enhanced by means of inviting experts on this issue, motivating nursing personnel to practise caring presence within the nursing profession.

**Regarding the fifth emergent theme: What caring presence is not**

- Hospital and nursing management should identify and address barriers that hinder the practice of caring presence by providing adequate resources, both human and monetary, to foster the practice of caring presence.
- Depersonalisation of patients should be recognised and seriously addressed by nursing management by means of implementing a system where nursing personnel who treat patients in an unethical, uncaring way (as objects) receive negative reports and warnings.

### Recommendations for nursing research

- Research on the relationship between passion for the nursing profession and the practice of caring presence.
- Research on PNs’ lived experience of practising caring presence in the private healthcare sector in South Africa.

## Conclusion

The findings of this qualitative, descriptive and phenomenological study can be used to expand and support the existing literature regarding the practice of caring presence. Consequently, the rich information and insight gained from the lived experiences of the PNs in this study add to the nursing body of knowledge, specifically regarding the understanding of the concept of caring presence from a South African point of view.
